# Supplementary material for: Cellular SPION Uptake and Toxicity in Various Head and Neck Cancer Cell Lines
Source: Nanomaterials (Basel). 2021 Mar 13;11(3):726. doi: 10.3390/nano11030726 (PMC7999062; doi:10.3390/nano11030726)
Supplement: Supplementary file 1 [file nanomaterials-11-00726-s001.pdf]

nanomaterials

Supplementary materials

Cellular SPION uptake and toxicity in various head and neck cancer cell lines

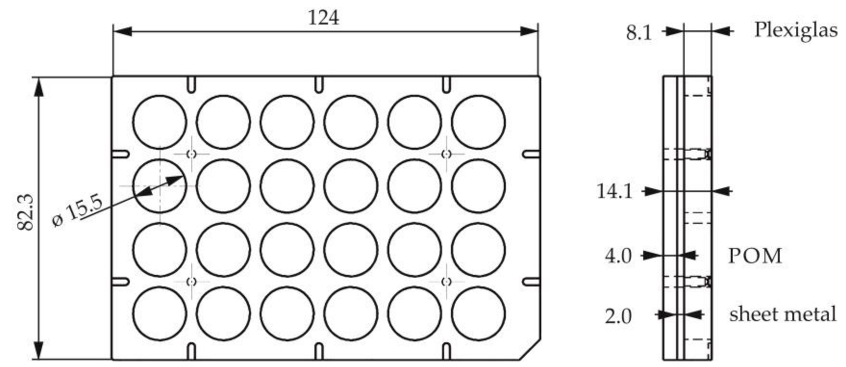

Figure S1. Design of the magnetic plates. The 3-layer construction of the magnetic plates consists of a top plate made of "Plexiglas GS" with 4 x 6 holes for round magnets (15 mm x 8 mm, Neodym N42), a middle plate of magnetic v2a sheet metal, and a bottom plate of 4 mm POM. The magnetic plates exert a maximum magnetic force of 380 mT on the bottom of the cell culture plate placed on the magnetic plate. Abbreviations: POM; polyoxymethylene.

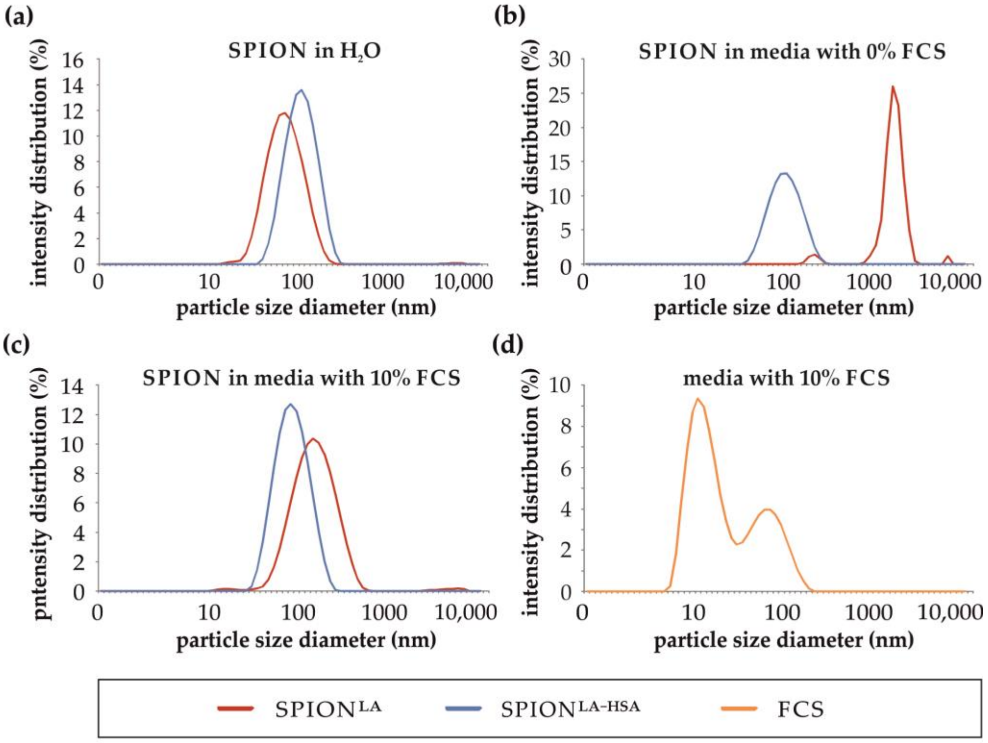

Figure S2. Size measurements of $SPION^{LA}$ and $SPION^{LA\text{-}HSA}$ by dynamic light scattering (DLS). Hydrodynamic diameter were determined after dilution of SPION stock solutions in (**a**) ultrapure water, (**b**) media without FCS supplement, and (**c**) media with 10% FCS. (**d**) Measurement of SPION-free media with 10% FCS. Abbreviations: SPION, superparamagnetic iron oxide nanoparticles; $SPION^{LA}$, lauric acid-coated SPIONs; $SPION^{LA\text{-}HSA}$, lauric acid- and human serum albumin-coated SPIONs; FCS, fetal calf serum.

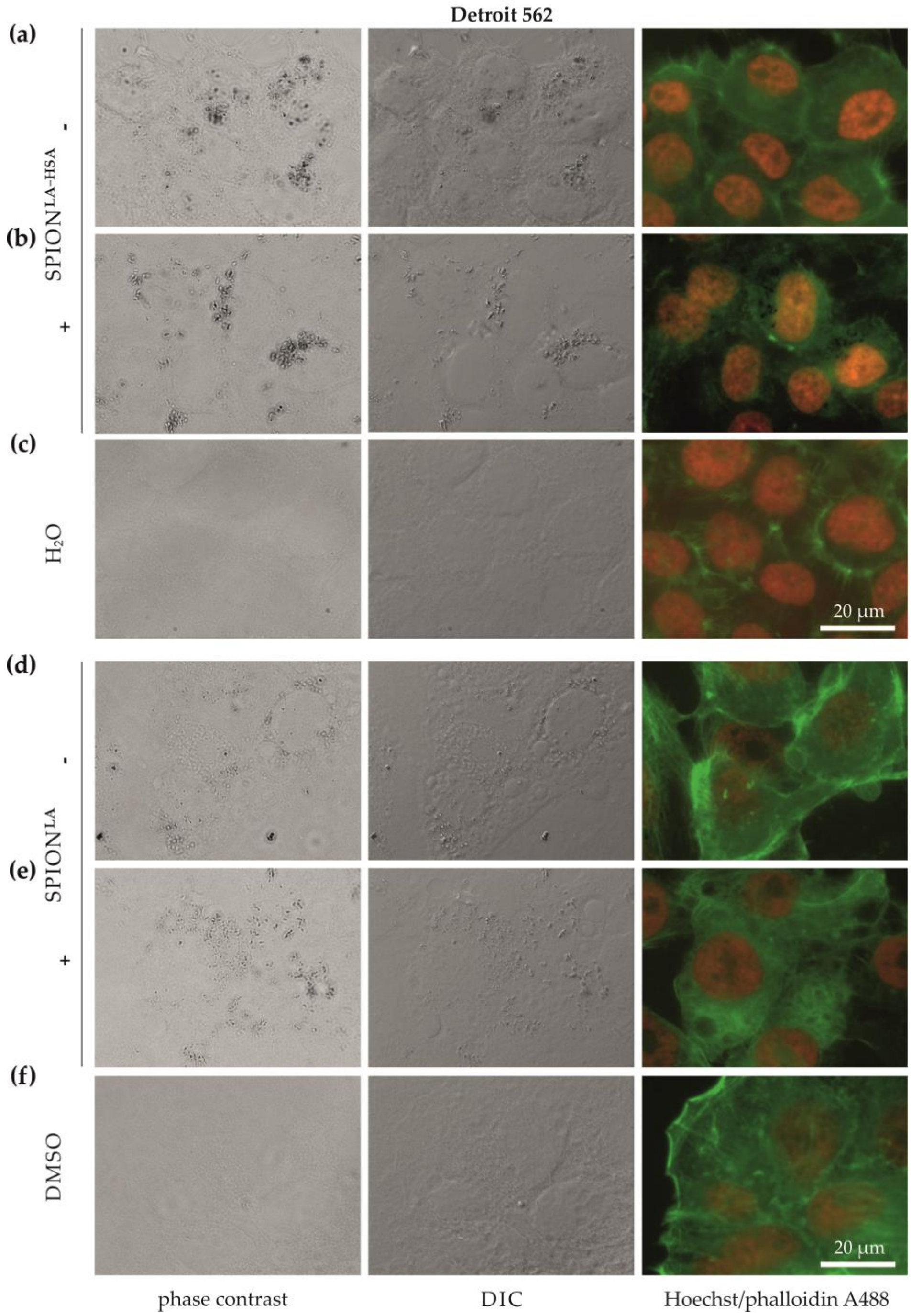

Figure S3. Optical imaging of Detroit 562 cells incubated with SPIONs in presence and absence of external magnets. Cells were treated with (**a, b**) $SPION^{LA\text{-}HSA}$ and (**d, e**) $SPION^{LA}$ (100 µgFe/mL) in the presence (+) or absence (-) of a magnet or with the corresponding amount of (**c**) H_2O or (**f**) DMSO (final concentration of 2%) for 48 h and visualized by phase contrast (first column), DIC (middle column) and fluorescent staining of nuclei (Hoechst 33342, red) and actin cytoskeleton (Alexa Fluor 488 Phalloidin, green) (last column). Abbreviations: SPION, superparamagnetic iron oxide nanoparticles; $SPION^{LA}$, lauric acid-coated SPIONs; $SPION^{LA\text{-}HSA}$, lauric acid- and human serum albumin-coated SPIONs; DIC, differential interference contrast; DMSO, dimethyl sulfoxide.

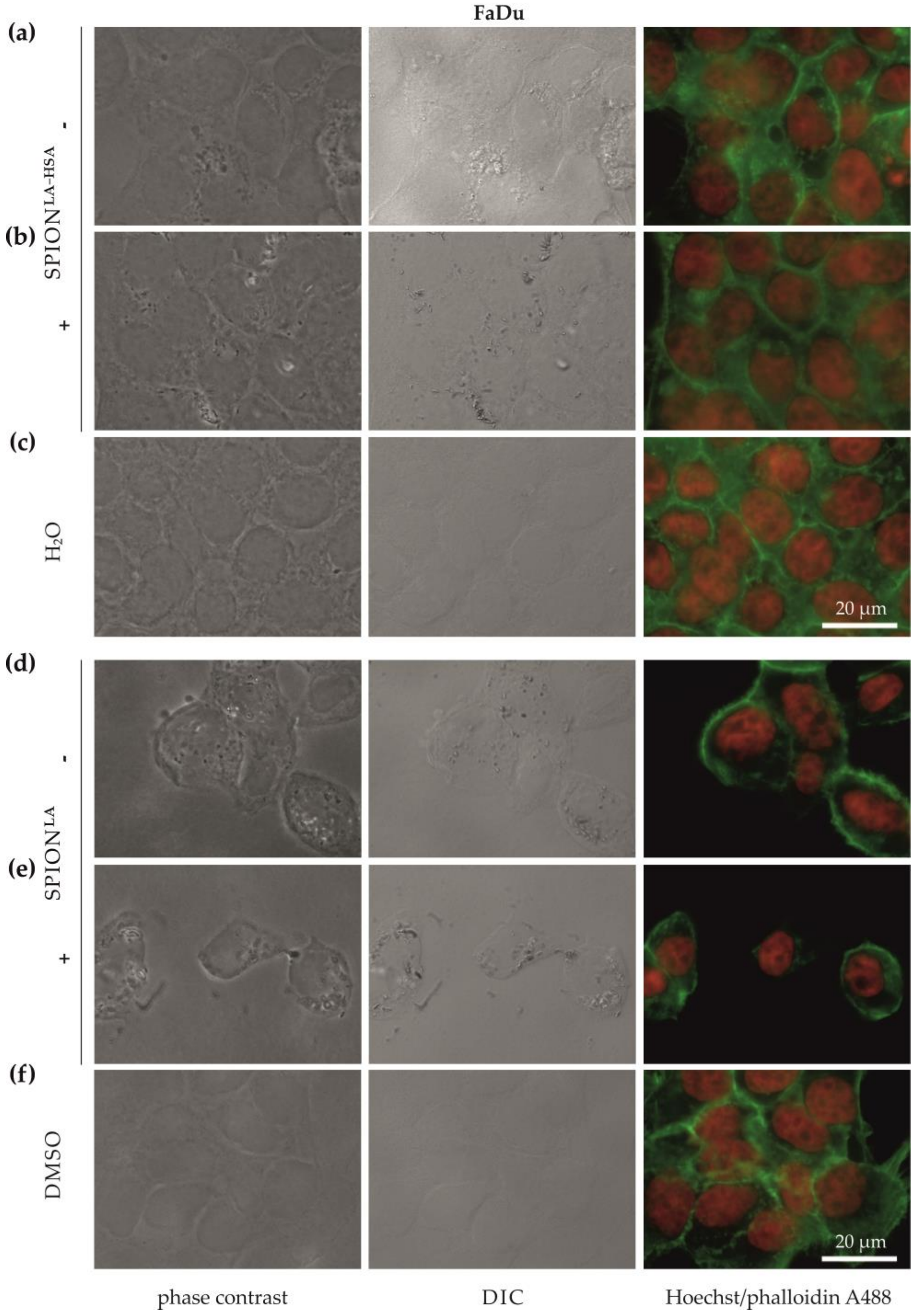

Figure S4. Optical imaging of FaDu cells incubated with SPIONs in presence and absence of external magnets. Cells were treated with (**a, b**) SPION^{LA-HSA} and (**d, e**) SPION^{LA} (100 µgFe/mL) in the presence (+) or absence (-) of a magnet, or with the corresponding amount of (**c**) H_2O or (**f**) DMSO (final concentration of 2%) for 48 h and visualized by phase contrast (first column), DIC (middle column) and fluorescent staining of nuclei (Hoechst 33342, red) and actin cytoskeleton (Alexa Fluor 488 Phalloidin, green) (last column). Abbreviations: SPION, superparamagnetic iron oxide nanoparticles; SPION^{LA}, lauric acid-coated SPIONs; SPION^{LA-HSA}, lauric acid- and human serum albumin-coated SPIONs; DIC, differential interference contrast; DMSO, dimethyl sulfoxide.

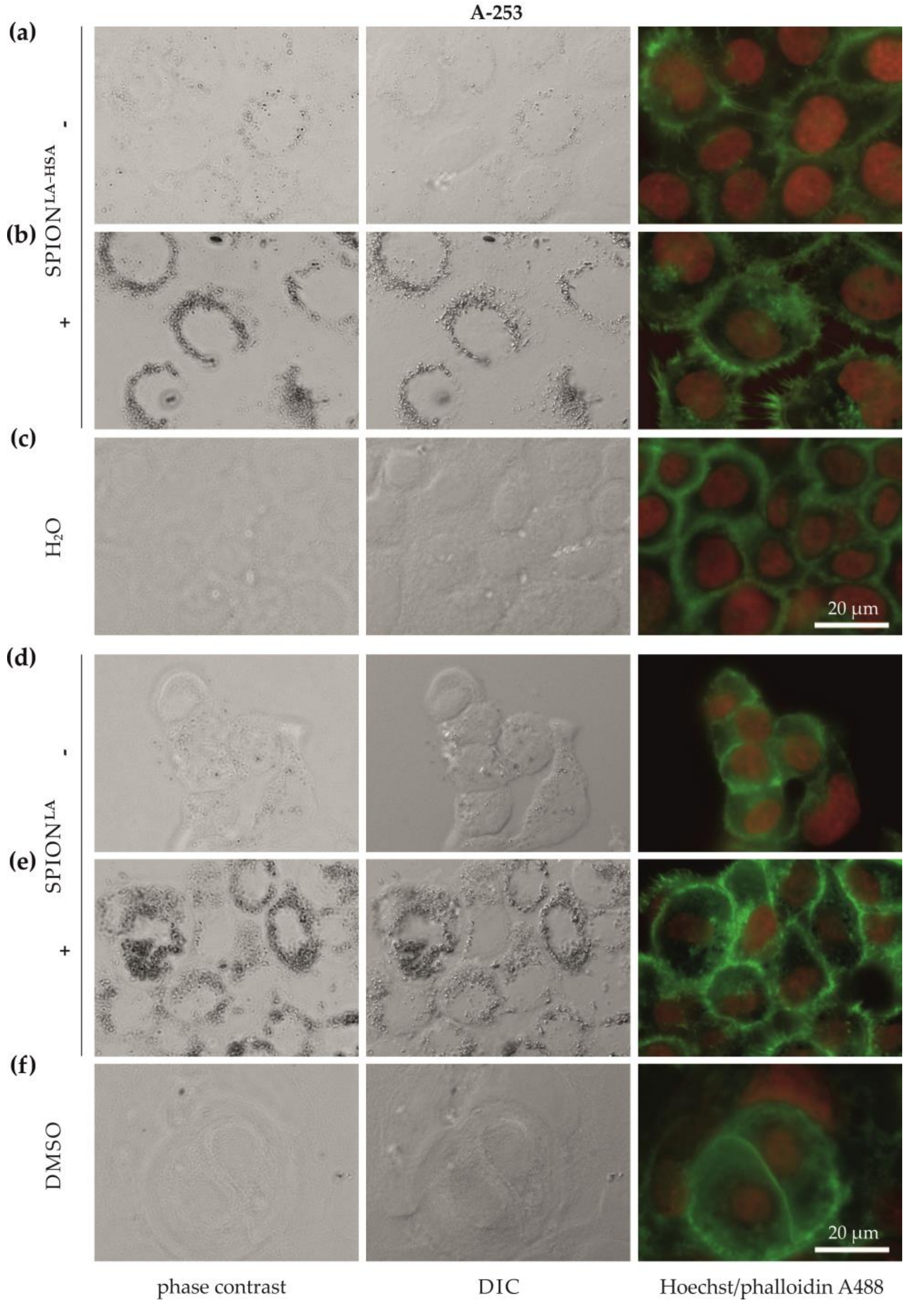

Figure S5. Optical imaging of A-253 cells incubated with SPIONs in presence and absence of external magnets. Cells were treated with (**a, b**) SPION^{LA-HSA} and (**d, e**) SPION^{LA} (100 µgFe/mL) in the presence (+) or absence (-) of a magnet, or with the corresponding amount of (**c**) H_2O or (**f**) DMSO (final concentration of 2%) for 48 h and visualized by phase contrast (first column), DIC (middle column) and fluorescent staining of nuclei (Hoechst 33342, red) and actin cytoskeleton (Alexa Fluor 488 Phalloidin, green) (last column). Abbreviations: SPION, superparamagnetic iron oxide nanoparticles; SPION^{LA}, lauric acid-coated SPIONs; SPION^{LA-HSA}, lauric acid- and human serum albumin-coated SPIONs; DIC, differential interference contrast; DMSO, dimethyl sulfoxide.

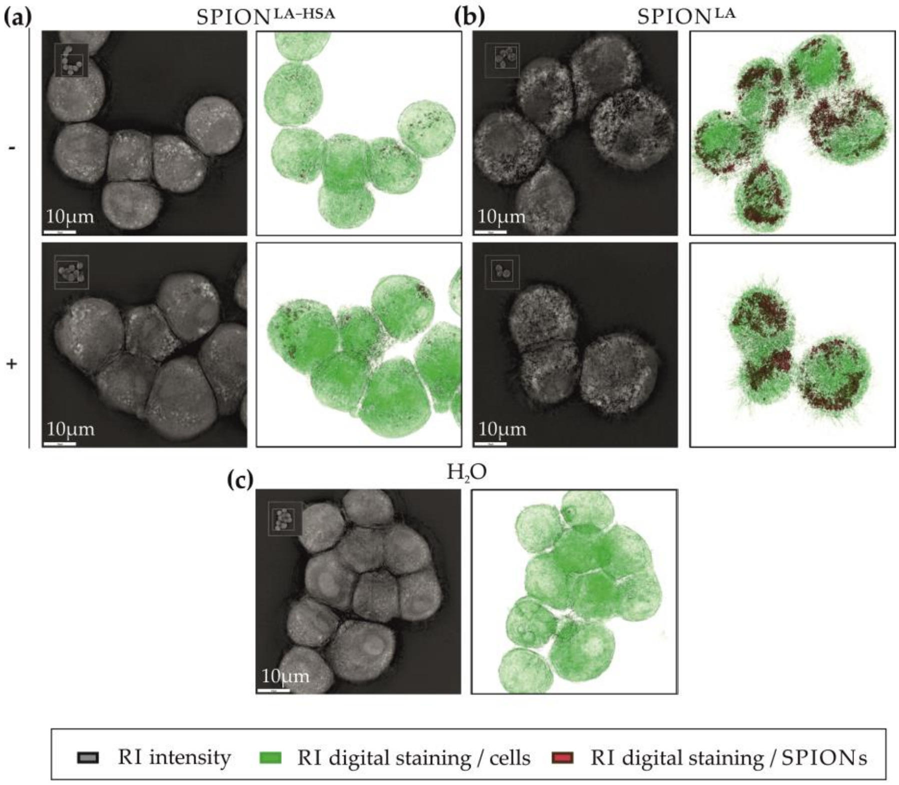

Figure S6. Holotomographic imaging of Detroit 562 cells after incubation with SPIONs. Cells were incubated with SPIONs in the presence and absence of an external magnet. Cells were treated with unlabeled (**a**) SPION$^{LA\text{-}HSA}$ and (**b**) SPION^{LA} (100 μgFe/mL) in the presence (+) or absence (-) of a magnet or with the corresponding amount of (**c**) H_2O for 48 h and visualized by differences in refractive index. The 2D black and white images depict X-Y sections of the 3D RI composition. The 3D RI hologram is digitally colored, showing structures with low RI (cell nuclei, nucleoli and cytoplasm) in green and high RI (SPION clusters) in reddish-brown. The colored images show the top view of the 3D holograms. Abbreviations: SPION, superparamagnetic iron oxide nanoparticles; SPION^{LA}, lauric acid-coated SPIONs; SPION$^{LA\text{-}HSA}$, lauric acid- and human serum albumin-coated SPIONs; RI, refractive index.

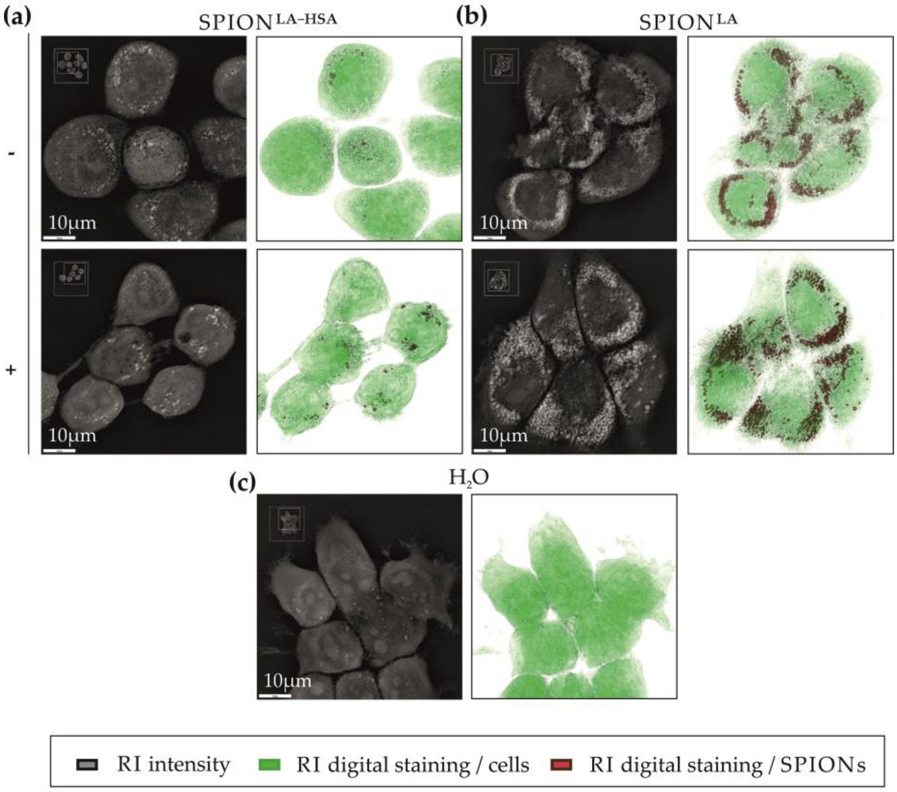

Figure S7. Holotomographic imaging of FaDu cells after incubation with SPIONs. Cells were incubated with SPIONs in the presence and absence of an external magnet. Cells were treated with unlabeled (**a**) $SPION^{LA\text{-}HSA}$ and (**b**) $SPION^{LA}$ (100 µgFe/mL) in the presence (+) or absence (-) of a magnet or with the corresponding amount of (**c**) H_2O for 48 h and visualized by differences in refractive index. The 2D black and white images depict X-Y sections of the 3D RI composition. The 3D RI hologram is digitally colored, showing structures with low RI (cell nuclei, nucleoli and cytoplasm) in green and high RI (SPION clusters) in reddish-brown. The colored images show the top view of the 3D holograms. Abbreviations: SPION, superparamagnetic iron oxide nanoparticles; $SPION^{LA}$, lauric acid-coated SPIONs; $SPION^{LA\text{-}HSA}$, lauric acid- and human serum albumin-coated SPIONs; RI, refractive index.

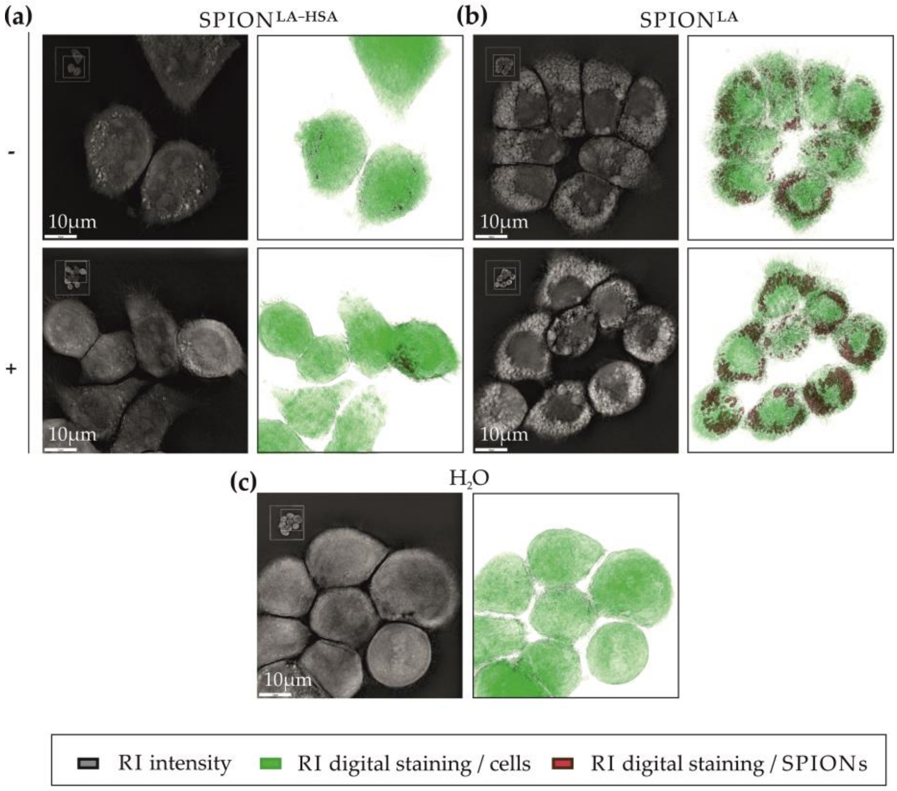

Figure S8. Holotomographic imaging of A-253 cells after incubation with SPIONs. Cells were incubated with SPIONs in the presence and absence of an external magnet. Cells were treated with unlabeled (**a**) SPION^{LA-HSA} and (**b**) SPION^{LA} (100 µgFe/mL) in the presence (+) or absence (-) of a magnet or with the corresponding amount of (**c**) H_2O for 48 h and visualized by differences in refractive index. The 2D black and white images depict X-Y sections of the 3D RI composition. The 3D RI hologram is digitally colored, showing structures with low RI (cell nuclei, nucleoli and cytoplasm) in green and high RI (SPION clusters) in reddish-brown. The colored images show the top view of the 3D holograms. SPION, superparamagnetic iron oxide nanoparticles; SPION^{LA}, lauric acid-coated SPIONs; SPION^{LA-HSA}, lauric acid- and human serum albumin-coated SPIONs; RI, refractive index.

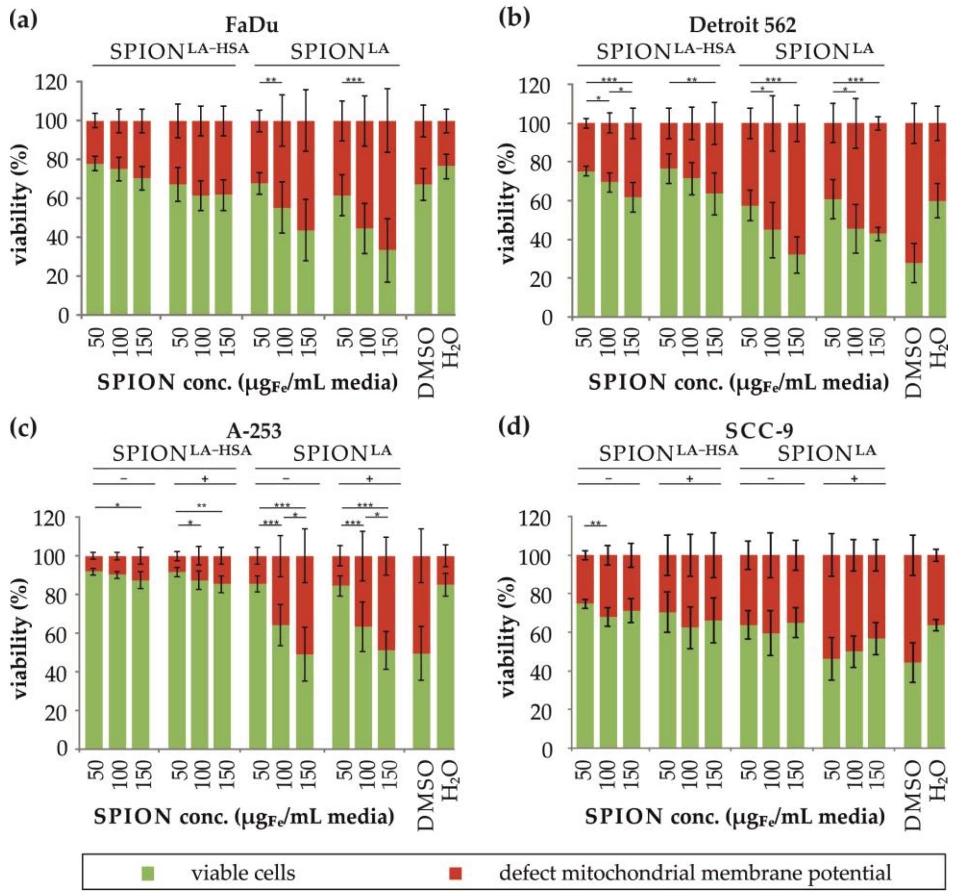

Figure S9. Changes in the mitochondrial membrane potential (mmp) after SPION treatment in the presence or absence of an external magnet. Cell viability was determined by DiIC1(5) staining and analyzed by flow cytometry. The number of cells with intact mmp (DiIC1(5) positive) and cells with defective mmp (DiIC1(5) negative) are shown for (**a**) FaDu, (**b**) Detroit 562, (**c**) A-253, and (**d**) SCC-9 after treatment with SPION^{LA} or SPION^{LA-HSA} in concentrations 50 µg/ml, 100 µg/mL and 150 µg/mL with and without a magnet (-/+). Toxicity controls contain 2% DMSO and negative controls the equivalent amount of H_2O. Data are expressed as the mean ± standard deviation (n=4 with technical quadruplicates). Statistical significance related to the percentage of cells with intact mmp are indicated by *, ** and ***. The respective confidential intervals are $p \leq 0.05$, $p \leq 0.001$ and $p \leq 0.0001$ and were calculated using Student's t-test analysis. Abbreviations: SPION, superparamagnetic iron oxide nanoparticles; SPION^{LA}, lauric acid-coated SPIONs; SPION^{LA-HSA}, lauric acid- and human serum albumin-coated SPIONs.

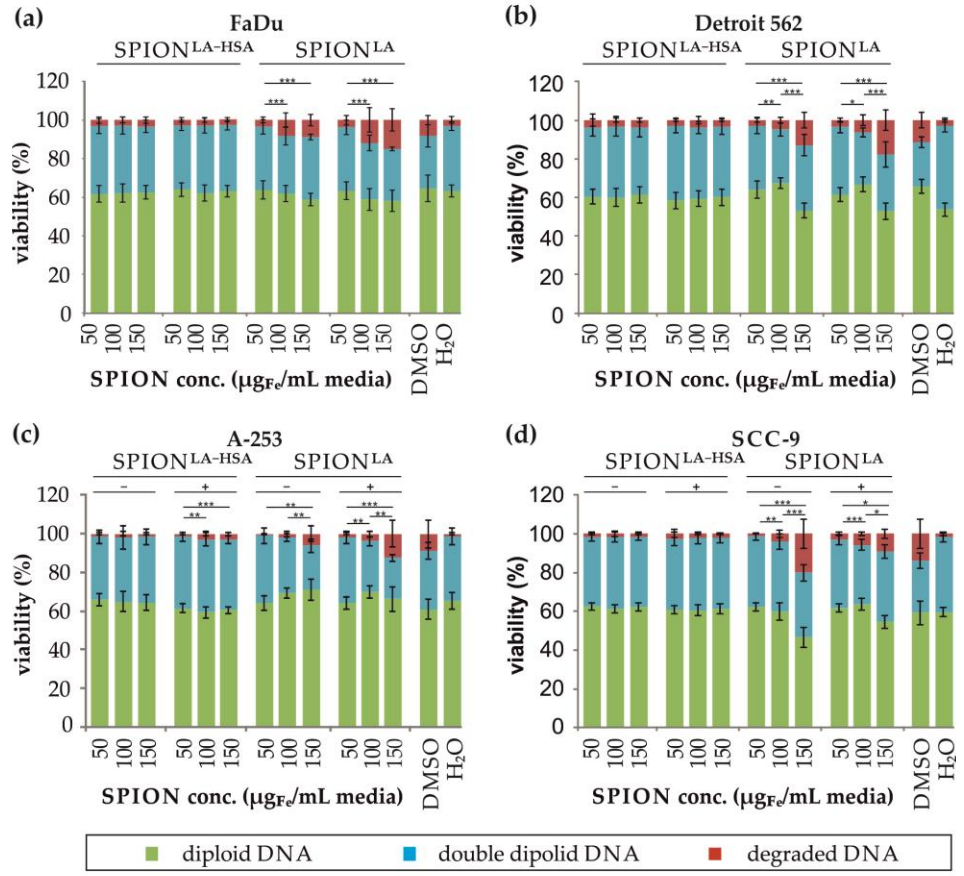

Figure S10. DNA degradation and cell cycle analysis by propidium iodide-triton X (PIT) staining. The amount of diploid DNA (G1-phase), double diploid DNA (G2-phase), and degraded DNA in FaDu (**a**), Detroit 562 (**b**), A253 (**c**) and SCC-9 (**d**) after treatment with SPIONs in the concentrations 50, 100 and 150 μg_{Fe}/mL without or on external magnets (-/+) was determined by flow cytometry after PIT staining. Controls contained 2% DMSO or the equivalent amount of H_2O. Data are expressed as the mean standard deviation (n=3 with technical triplicates). Statistical significance related to the percentage of cells with degraded DNA are indicated by *, ** and ***. The respective confidential intervals are $p \leq 0.05$, $p \leq 0.001$ and $p \leq 0.0001$ and were calculated via Student's t-test analysis. Abbreviations: SPION, superparamagnetic iron oxide nanoparticles; $SPION^{LA}$, lauric acid-coated SPIONs; $SPION^{LA\text{-}HSA}$, lauric acid- and human serum albumin-coated SPIONs; DMSO, Dimethyl sulfoxide.

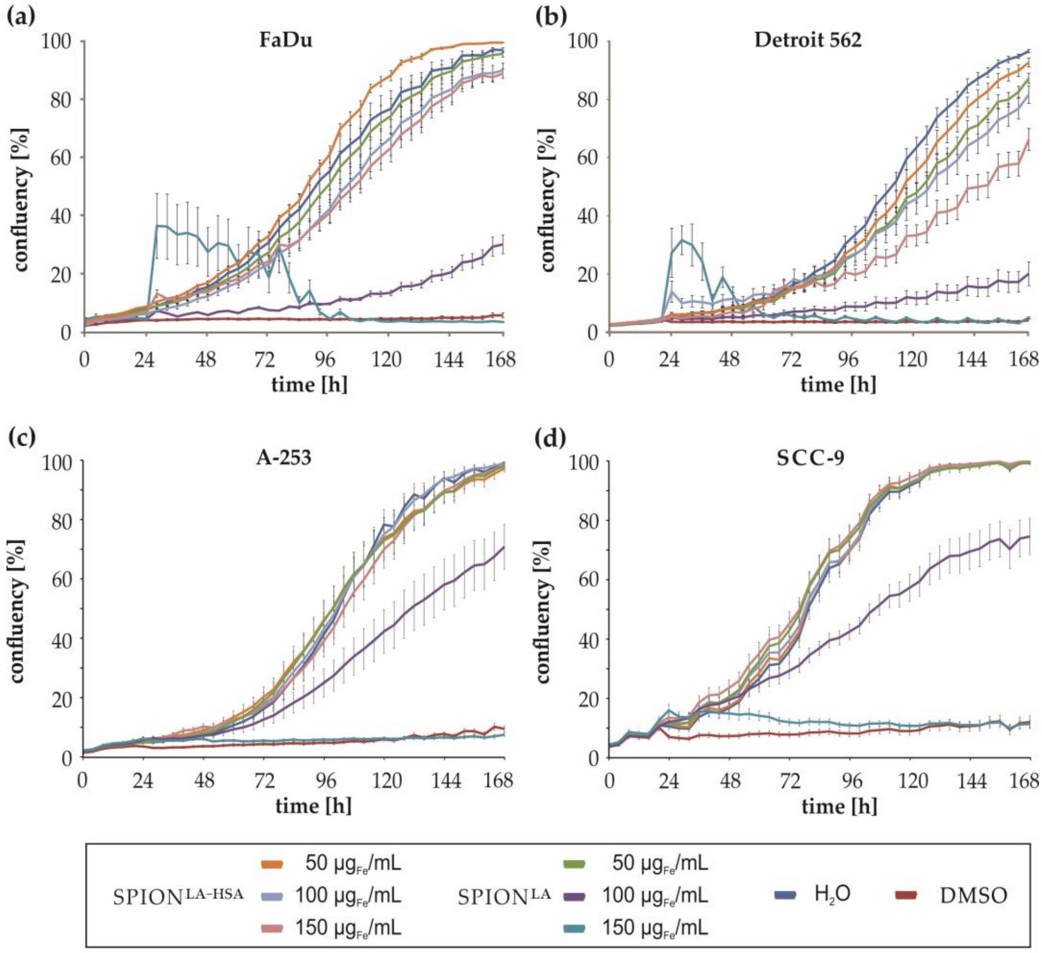

Figure S11. Growth curves of head and neck tumor cell lines generated by optical live cell imaging. Growth curves of the cell lines FaDu (**a**), Detroit 562 (**b**), A253 (**c**) and SCC-9 (**d**) were produced by monitoring the change in cell confluency after the addition of SPION^{LA} or SPION^{LA-HSA} at concentrations 50, 100 and 150 µg$_{Fe}$/mL cell culture medium. The positive control contains 2% DMSO, the negative control H_2O instead of the SPION suspension. Data are expressed as standard error of the mean (n=4 with 8-fold replicates). Abbreviations: SPION, superparamagnetic iron oxide nanoparticles; SPION^{LA}, lauric acid-coated SPIONs; SPION^{LA-HSA}, lauric acid- and human serum albumin-coated SPIONs; DMSO, Dimethyl sulfoxide.

Table S1. Cellular SPION uptake of SPIONs in different cells. Shown are the results obtained from head and neck tumor cells and breast cancer cells (indicated by italics) together with endothelial cells investigated in a previous study [1]. All cell lines were incubated with 50 µg/mL $SPION^{LA}$ or $SPION^{LAHSA}$ for 48 h and analyzed by AES. Abbreviations: SPION, superparamagnetic iron oxide nanoparticles; $SPION^{LA}$, lauric acid-coated SPIONs; $SPION^{LA-HSA}$, lauric acid- and human serum albumin-coated SPIONs.

cell line	$SPION^{LA}$ (pg/cell)	$SPION^{LA-HSA}$ (pg/cell)
BT-474	1.1 ± 0.3	1.0 ± 0.3
T-47D	1.1 ± 0.6	0.6 ± 0.2
MCF7	0.7 ± 0.2	0.5 ± 0.2
MDA-MB-231	1.1 ± 0.3	0.6 ± 0.2
FaDu	1.3 ± 0.4	0.5 ± 0.3
Detroit 562	1.1 ± 0.3	0.4 ± 0.2
A-253	1.1 ± 0.3	0.4 ± 0.1
SCC-9	1.7 ± 0.5	0.5 ± 0.2
HUVEC	3.0 ± 0.6	0.7 ± 0.2

References

1. Poller, J.M.; Zaloga, J.; Schreiber, E.; Unterweger, H.; Janko, C.; Radon, P.; Eberbeck, D.; Trahms, L.; Alexiou, C.; Friedrich, R.P. Selection of potential iron oxide nanoparticles for breast cancer treatment based on in vitro cytotoxicity and cellular uptake. *International journal of nanomedicine* **2017**, *12*, 3207-3220, doi:10.2147/IJN.S132369.
